# A Case of Morbus Cœruleus, Attended with Infiltration of Blood under the Hairy Scalp

**Published:** 1829

**Authors:** William M. Voris

**Affiliations:** West Union, Ohio


Art. VIII.—*A case of Morbus Cœruleus, attended with infiltration of blood under the hairy scalp.*—By Dr. William M. Voris, of West Union, Ohio.

The subject of the natural defect in the organization of the heart, constituting the disease before us, is a little girl aged nine years.

She is about the ordinary size for one of her age, and her general health is as good, as could be expected in such a case; all her functions being commonly performed well, excepting the circulation. The whole surface of her body presents a purple or bluish colour more or less deep, and is sometimes more so than at others. The sclerotic coat of her eyes is deeply tinged with dark red blood, her eyelids, lips and ears, are of a deep purple hue; her fingers and toes of the same, and their extremities much enlarged. She is extremely sensible to external impressions; her respiration is constantly more or less impeded; her pulse is always irregular, and manifests to the fingers a tremulous action; sometimes a kind of angry motion, as if the heart was labouring preternaturally; and these peculiarities are aggravated by the least exertion or stimulus acting on the body or mind. She, nevertheless, possesses an acuteness of perception and understanding, rather above her age, and does not exhibit that hebetude, and deficient energy, which are generally described by authors, as attending this disease.

She has generally enjoyed the amusements and sports of her brothers and sisters; though she could take no *active* part in their exercises; but followed them slowly from one place to another, when they were playing, and appeared to wish she could participate in their operations. She is so sensible that exercise always produces sensations of anguish, that she has not for several years, dared to do any thing that would excite her system, unless compelled by unavoidable circumstances.

Her parents told me that several physicians had predicted that their little daughter would not survive the age of fourteen years, and that it was likely she would expire suddenly.—This her little brothers and sisters overheard, and told her, the doctor had said, she would not live long. Poor little Jane heard it with serenity, but not without some degree of sorrow and anxiety.

Her general health, as mentioned above, has been moderately good, but from the time she was capable of expressing herself intelligibly, she has occasionally complained of severe pain in her forehead and temples, which was sometimes excruciating. These paroxysms commonly continued from twelve to eighteen hours, and left her in her usual health.

About the 15th of July last, she was attacked with this pain in her forehead and left temple, which continued and increased to an alarming degree. On the 25th, the family, residing about 15 miles from town and not before having had a physician, became alarmed, and sent for my partner, Dr. Wilson, or myself. On the evening of this day, I visited her, about ten days after her attack. I found the scalp swollen to an alarming degree over every part of the head, excepting the os occipitis. The forehead, eyebrows and lids, partook of this swelling to such a degree that it was difficult to separate the eyelids so as to expose the eyes. The eyes also were swollen and their vessels loaded with blood. Although, as her parents told me, the febrile symptoms had considerably abated, during the two days previously, her pulse was still excited, and attended with the peculiarities above mentioned. Her tongue was livid and dry, skin harsh and husky, urine scarce and lateritious, and thirst continual. The stomach sympathized, and was affected with loathing of food and nausea. She was no wise able to hold up her head, unless supported, and appeared inclined to lie with it low. Her sufferings were at this time extreme. Her pain was in paroxysms, which lasted ten or fifteen minutes, and came on as often as every half hour; in the interval she commonly lay composed, and sometimes slept. Although she laboured under so much vascular excitement, with determination to the brain, and suffered so much pain, that she often wept with agony, she had no mental derangement, nor exhibited the least evidence of oppressed brain.

When I examined her head, I found that the skin all over the swollen part, had assumed the deep purple colour, in perfect keeping with the extreme parts mentioned before. On the first examination I took it to be a case of hydrocephalus externus; but upon examining more particularly, my views were changed. On applying the fingers to different parts of the head, a fluctuation was unquestionable, and the scalp was evidently detached from its cellular connexion with the pericranium, in every part, excepting behind. She had received no injury, neither had previous disease existed in this part; consequently, suppuration could not reasonably be suspected.

The poor little sufferer's case appeared to me almost hopeless. She was in extreme pain, and required immediate attention; but with regard to the medical treatment, I was at a loss to decide on a judicious plan. After using various means, calculated to restore the lost equilibrium, and invite the force of the circulation to the skin and extremities, so as to unload the vessels of the head; I resolved on trying the application of a large blistering plaister to the nape of the neck. This was applied the same evening, about four hours after I first saw her. I gave her at the same time 10 grs. of the protochloride of mercury, succeeded by pulv.

jalapi et supertart. pot. a a gr. xvi. a few hours afterwards. By 8 o'clock next morning the cathartic had operated very freely on the bowels, the blister had drawn well, and was filled with a transparent and very tenacious jelly, which could be cut into slices with a knife. By this time her pain was considerably mitigated, and her eyes less inflamed, yet the tumefaction was not sensibly diminished.

These means promising no more than a temporary mitigation of pain, I determined at 9 o'clock this morning to evacuate the fluid by making a puncture in the temple. On taking a view of the circumstances of the case in the aggregate, I thought there could be no reasonable hope of a final recovery; but concluded this operation promised to relieve the severity of the pain, by taking off the distention and compression, produced by so large an accumulation of fluid, and consequently to mitigate the violence of the other symptoms.—When I withdrew the lancet, after making a free opening in the most dependent part on the left temple, there gushed out, *in pleno rivo*, a black fluid with a very slight tinge of red, which was doubtless dissolved blood, which had been extravasated, by a morbid action of the vessels. The fœtor was considerable, yet not more so, than might be expected in a fluid of this kind, which had been extravasated for several days, and of course had lost its vitality. After a pint and a half was discharged, it sudddenly ceased flowing; and fearing that syncope would take place, (of which there were already symptoms) from the sudden removal of the stimulus of distention, I closed the orifice, and placed her in a situation as favourable as possible for rest. In a very few minutes, the little sufferer expressed more ease than she had experienced since the beginning of the attack.

I now directed the parents of the child to encourage the discharge of the fluid, by opening the orifice two or three times a day, until all should be evacuated; and at the same time applied, an equally compressing bandage as firmly over the head as she could bear. As there still appeared to be a considerable determination to the head, I directed pulv. jalap. and crem. tart. each 15 grs. to be given every twenty four hours. I also advised sinapisms to be applied to the extremities, occasionally, and friction with hot brine to the surface of the body generally. A light and spare diet, was likewise enjoined.

On the 30th of this month, I saw my patient again. The parents informed me, that considerably upwards of half a gallon of fluid had been discharged, since I saw her before; and that the febrile excitement had entirely left her soon after my departure. She had been for the most part free from pain, and had rested well at night. The orifice in the temple had closed, there had been no discharge for two days, and the fluid was fast accumulating.

31st. The patient rested poorly last night, and the swelling is nearly as great as ever this morning. I immediately determined to make an opening, in the right temple, which was done, and from this was discharged about a pint of fluid, with considerably more of the reddish tinge than the former.—Shortly after this opening was made, the orifice made at my first visit, broke open spontaneously and discharged about the same quantity. From the two orifices, nearly all the fluid was evacuated, and the scalp lay loose and flabby over the cranium; and was entirely detached from the frontal, temporal and parietal bones.

The symptoms appearing so much more favourable at this time, than at first, I was encouraged to prescribe still further. Accordingly I had a cap of sheet lead prepared, and having introduced a tent into each orifice, and applied the above mentioned bandage, I put on the cap, and bound this on with another bandage. I directed these bandages to be removed morning and evening, and the head to be rubbed well with a strong volatile liniment. The jalap and crem. tartar to be continued, also the frictions with hot brine, and a liberal diet, if her stomach would receive it.

Aug. 4th. Received a letter from the father of our patient to day, in which he writes as follows—

"Jane has (I think) mended daily since you were here. She is now able to set up occasionally in her crib, and talk a little to those around her. The orifices have dischsrged at different times, and the fluid is neither so thick nor black as formerly. The blackness has in a great measure left her eyes; she can open them about half way, and keep them so when the room is darkened. The cream of tartar and jalap, have not been so effectual as at first, and we have sometimes to assist them by injections."

Aug. 5th. Sent by a messenger this morning the following prescription. Continue the lead cap and bandage to the head with the liniment: also the friction with brine. Discontinue the jalap and cream tartar, and use two or three of the following pills every night. ℞. Blue mercurial mass, aloes and squills a. a. one grain, mixed and made into one pill; of the sol. sulph quinine, half a teaspoonful four or five times daily, with 6 drops of elix. vitriol. Apply to the eyes occasionally a few drops of the following collyrium. ℞: Sulph. zinc. Acet. plumb. a. a. 1 gr. aquæ distillat. ℥j. misce. To which add 8 drops of laudanum.

Aug. 11th. The father same to town to day, and says Jane is mending daily; has a good appetite, and is able to set up frequently. The swelling has entirely left her head, excepting on one temple which appears to contain about an ounce of fluid. The orifice on that side has healed, and the other is discharging pus. The inflammation has almost left her eyes, and the deep purple colour of the extreme parts has become considerably lighter; the ends of her fingers are comparatively smaller than since her birth, and she is evidently gaining flesh and strength every day. The sol. sulph. quin. et acid. vit. appeared to increase the excitement, and after giving them a few times, he discontinued them.

As these medicines now appeared to be admissible, he was directed to resume their administration.

I received no further information respecting this interesting case, until the 25th of September, when I was called to visit a patient in the same family. I found little Jane enjoying a state of health and strength, considerably better than she was used to formerly.

Her parents told me she was more fleshy than she had ever been; that she had a good appetite, was lively and cheerful, and used no medicine excepting an occasional laxative. The scalp had united again as firmly as before her illness. She still, however, had all the symptoms characteristic of her congenital disease.

*December*, 1829.

